# Safe water treatment practices: A qualitative study on point-of-use chlorination in Nigeria

**DOI:** 10.7189/jogh.14.04178

**Published:** 2024-09-13

**Authors:** Ifeoma Idigbe, Michelle Cherian, Abideen O Salako, Babatunde Adewale, Babatunde L Salako, Elisa M Maffioli

**Affiliations:** 1 Nigerian Institute of Medical Research, Lagos, Nigeria; 2 Development Innovation Lab, University of Chicago, Chicago, Illinois, USA; 3 University of Ibadan, Ibadan, Nigeria; 4 University of Michigan, Ann Arbor, Michigan, USA

## Abstract

**Background:**

25% of the world’s population does not have access to safely managed drinking water. Point-of-use chlorination is a safe, inexpensive, and effective strategy to improve water quality and child health. We aimed to understand safe water treatment practices and the feasibility and acceptability of point-of-use chlorination in Nigeria.

**Methods:**

Between November 2022 and January 2023, six focus group discussions were conducted with pregnant women and mothers of children aged <5 years. Semi-structured, in-depth interviews were conducted with women, health facility workers, shopkeepers or pharmacy attendants, and water point owners. Data were collected and analysed using the socio-ecological model of health framework. Four themes were identified.

**Results:**

Theme 1: water culture – there was little knowledge about the need to treat drinking water since it was considered safe and high-quality, and there was low knowledge about point-of-use chlorination. Theme 2: improving the quality of life – there were reports of child diarrhoea and lost pregnancies. Yet, most respondents did not link maternal and child health to drinking water. Theme 3: getting support – stakeholders were interested in point-of-use chlorination and agreed to link the programme to health workers already providing care for pregnant women and children. Theme 4: advocating for safe water – educating communities on the importance of safe drinking water and integrating services within communities is key.

**Conclusions:**

Safe drinking water and interventions such as point-of-use chlorination improve the quality of life. Yet, given the perception that existing water is safe and the lack of knowledge about the value of chlorination, awareness is the priority for change. This study demonstrated the potential for point-of-use chlorination if well-integrated and supported by different stakeholder groups.

Approximately 2.2 billion people worldwide, about 25% of the world’s population, still do not have access to safely managed drinking water [1]. For those who live where large-scale investment in water infrastructure is lacking, point-of-use chlorination at the household level could be an alternative to realise the health benefits of safe drinking water [2]. Chlorination is safe, inexpensive, and effective in inactivating most pathogens that cause diarrhoea [3]. A recent meta-analysis combining data from several randomised evaluations finds that water treatment reduces child mortality by around a quarter [4]. Yet, the burden of fetching and treating water remains on women and children [5,6].

There is large descriptive evidence evaluating households’ water treatment practices and their associated factors in African countries [7–11], indicating that previous knowledge of water treatment, income and educational status are predictors of household use of water treatment, including chlorination. In addition, qualitative studies explored factors influencing household water treatment practices [12], as well as facilitators and barriers to the uptake of water, sanitation and hygiene interventions [13–16]. Finally, there is empirical evidence on the impact of safe water treatment on diarrhoea [17,18] and mortality [4], especially for chlorination. Yet, we still lack a better understanding of the feasibility and acceptability of safe water treatment practices, particularly point-of-use chlorination, in countries such as Nigeria, where there is a lack of knowledge about it.

In this study, we aimed to understand safe water treatment practices in three states in Nigeria (Kano, Lagos, and Ogun), taking into account the perspectives of different stakeholders. We targeted both the primary beneficiaries (pregnant women and mothers of children aged <5 years) and relevant stakeholders within the community, such as health facility workers, shopkeepers or pharmacy attendants, and water point owners, to comprehensively understand the feasibility and acceptability of safe water interventions, in particular point-of-use chlorination. By adopting a socio-ecological model of health framework, we attempted to identify barriers and facilitators to implementing point-of-use chlorination within the communities, thus shedding light on the potential for interventions supported by different stakeholder groups.

## METHODS

### Framework

**Figure 1:**
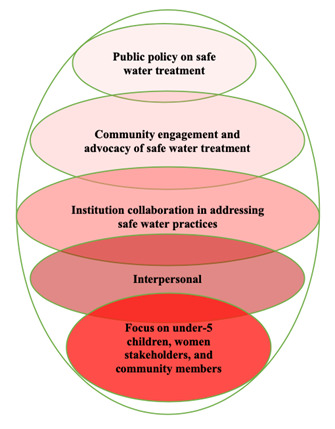
Adoption of the socio-ecological model of health framework. The figure describes the processes and strategies to explore safe water practices.

We adopted the socio-ecological model of health to explore safe water treatment practices and their potential impact on women and children aged <5 years [19–22]. The socio-ecological model of health is an approach that focuses on the interaction of systems and a multilevel conceptualisation [21,22]. It describes health as being affected by the interaction between individuals, the community and social institutions or environments [20,23,24]. A similar model has been used to explore the uptake of HIV treatment [25] or intermittent preventive treatment of malaria in pregnancy in Nigeria [26]. We adapted and used the socio-ecological model of health framework to understand the feasibility and acceptability of safe water interventions in Kano, Lagos, and Ogun states in Nigeria (**Figure 1**).

### Study setting

This study was conducted in health facilities and communities in Kano, Lagos, and Ogun states, more specifically in the following local government areas (LGAs): Kura, Tarauni, and Kano Municipal (Kano), Ijede, Surulere, and Yaba (Lagos), and Abeokuta North, Ado Odo Ota, Abeokuta South, and Ewekoro (Ogun). In Nigeria, 28.5% of people still do not have access to safely managed drinking water (33.8% in Kano, 2.1% in Lagos, and 25.2% in Ogun) [27]. Child morbidity and mortality are also very high in the country, with Kano having one of the highest mortality for children aged <5 years (148 per 1000 births), followed by Ogun (85 per 1000 births) and Lagos (15 per 1000 births) [27].

### Participant selection

In this study, we targeted pregnant women and mothers of children aged <5 years and relevant stakeholders within the community (health facility workers, shopkeepers or pharmacy attendants, and water point owners). Primary health care centres (PHCs) served as the initial point of contact for recruiting the targeted population. Collaborating with health workers at these facilities allowed access to a sample of pregnant women and mothers of young children coming in for health services. We conducted interviews with a broader range of individuals to gain a comprehensive understanding of the community context. We interviewed health workers at the PHCs and selected communities close to them to interview other stakeholders. These included shopkeepers or pharmacy attendants, who may provide water treatment supplies, and water point owners, who manage community water access points. These individuals were selected near the PHCs used for initial participant recruitment. Pharmacists within the PHC were also asked to participate in the study if available.

### Data collection

We conducted focus group discussions (FGDs) to enable the exploration of shared experiences, perceptions, and social norms regarding safe water practices within the community. We facilitated group discussions with pregnant women and mothers of young children. A total of six FGDs with seven to eight women each were conducted in Kano and Lagos states, one per LGA in each state. FGDs were conducted among pregnant women and mothers of children aged <5 years who were selected from available women waiting for antenatal care at the PHC and were made up of heterogeneous groups to ensure proper socio-demographic representation and inclusion. The mothers who participated in the FGDs were given detergents and soap in Kano and Maggi spice packets in Lagos as appreciation for their time and participation. We decided not to conduct FGDs in Ogun state, the last state where data collection was implemented, since the interviews provided similar but more in-depth information than FGDs. Instead, we increased the number of individual interviews in Ogun state.

Further, we conducted in-depth semi-structured interviews (IDIs). We conducted semi-structured interviews with both the targeted population and stakeholders, which allowed for an in-depth exploration of personal experiences and perspectives while maintaining flexibility in addressing unforeseen topics. In each LGA in each state, we targeted a few individuals by type of stakeholders (women, health facility workers, shopkeepers or pharmacy attendants, and water point owners). We interviewed seven to 10 women in each LGA in Kano and Lagos and almost double in Ogun when available (where we did not conduct FGDs). We were able to interview between two and six other stakeholders in each LGA in each state, except for one LGA in Ogun (Table S1 in **Online Supplementary Document**).

Local experienced research assistants, MSc and PhD holders working as social and behavioural or public health scientists for over four years were employed in the three states to collect data. A call for candidates was put out at each LGA’s local universities and health centres. Those who responded were screened through interviews to evaluate their background, preparation and communication skills. The candidates who passed the screening process were selected and trained in qualitative data collection. Fifteen female research assistants (seven in Kano, four in Lagos, and four in Ogun) and five male research assistants (two in Lagos and three in Ogun) were recruited and trained for the study.

In each state, fieldwork activities lasted for about two weeks. The following activities were conducted between November 2022 and January 2023 (26 November to 2 December 2022, in Kano; 6–16 December 2022 in Lagos, and 22–31 January 2023 in Ogun): community mobilisations to ask permission at the PHC and health facility’s head (two days), research assistants’ selection and training (three to four days), FGDs and IDIs (three days) and note compilation (two days). Data were collected using semi-structured questionnaires from eligible participants who consented to participate. Participants in each LGA in each state were purposefully selected for the FGDs and IDIs. Research assistants explained the process of conducting the interviews and the consenting process. Participants were enlightened about the voluntary interviews and their rights to participate, declining initially or at any point during the interview. Participants who agreed to participate were given consent forms to read and sign. All interviews were conducted in comfortable and private waiting areas in the selected PHCs. Interviews were conducted in English and local dialects (Hausa and Yoruba). Interviews were conducted in the local dialects and were translated into English by the local research assistants who spoke the dialects. Unique identifiers were assigned to participants. The facilitators ensured participants could express themselves and share their opinions and experiences. The data collected were stored in recorders, and copies of the recordings were saved in encrypted storage devices for analysis.

### Data analysis

We followed [28] the six-phase framework for doing thematic analysis, which is a theoretically flexible method aimed at ‘developing, analysing and interpreting patterns across a qualitative data set’ [29]. The data were analysed by a team with expertise in qualitative research methods, and Consolidated Criteria for Reporting Qualitative Research (COREQ) guidelines were followed. The team (II and MC) repeatedly listened to the audio recordings extracted from the interviews to become conversant, transcribed them and linked them appropriately to the transcriptions [30,31]. In addition, the team held meetings to review and discuss the transcripts and field notes. After all the field notes were finalised, the research team created themes from the transcripts and notes and followed an inductive approach to coding the qualitative data. This approach involved creating codes from the data set [32]. In other words, we did not start with preconceived notions of the codes but allowed the narrative or theory to emerge from the data set. The coding process was done manually, extracting codes from the transcripts’ data set and assigning them into categories [33]. Our codes were essentially tags for allocating identified themes to the data compiled in the study. The codes were synthesised, reviewed and compared to reflect similar and overarching themes until saturation. Themes and sub-themes were developed, and verbatim quotes were extracted to support them [29,34–36].

## RESULTS

### Descriptive statistics

Across 10 LGAs and three Nigerian states, a total of 47 women participated in six FGDs, and 200 individuals (95 women, 35 health workers, 32 shopkeepers or pharmacy attendants, and 38 water point owners) participated in the IDIs (Table S1 in **Online Supplementary Document**). **Table 1** shows the demographics of pregnant women and mothers of children aged <5 years who participated in the FGDs. The average age of women was 27 years. Most women were 18–29 years old (n = 22) and had secondary-level education (n = 20). Most participants were engaged in elementary occupations such as selling food items or running other forms of petty businesses (n = 13) or more skilled occupations (n = 12). The majority (n = 27) had a child aged <5 years during the data collection. Regarding existing water practices, most women (n = 34) did not apply any treatment to their drinking water and instead reported either drinking sachet or borehole water. Only 13 reported having heard about chlorine, and six used it.

**Table 1:** Demographics of pregnant women and mothers of children aged <5 y who participated in the focus group discussions*

Demographics	Total (n = 47), n (%)
Age in years	
*<18*	1 (2.1)
*18–29*	22 (46.8)
*30–49*	9 (19.1)
*>50*	0 (0.0)
*Missing*	15 (31.9)
Level of education	
*No schooling*	1 (2.1)
*Primary*	5 (10.6)
*Secondary*	20 (42.6)
*Bachelors*	2 (4.3)
*Masters*	0 (0.0)
*Diploma*	4 (8.5)
*Missing*	15 (31.9)
Occupation	
*Student*	0 (0.0)
*Unemployed*	9 (19.1)
*Elementary occupations*	13 (27.7)
*Skilled occupations*	12 (25.5)
*Professional occupations*	1 (2.1)
*Missing*	12 (25.5)
Any child aged <5 y	
*Yes*	27 (57.4)
*No*	13 (27.7)
*Missing*	7 (14.9)
Water treatment	
*Boiling*	5 (10.6)
*Filtration*	2 (4.3)
*Chlorine*	1 (2.1)
*None*	34 (72.3)
*Missing*	5 (10.6)
Knowledge of chlorine	
*Yes*	13 (27.7)
*Missing*	34 (72.3)
Ever used chlorine	
*Yes*	6 (12.8)
*Missing*	41 (87.2)

*Number of observations are reported. Proportions are reported in parenthesis. Occupational groups are based on the International Classification of Occupations published by ILO in 2008 [37]. Missing figures are reported for each category when present.

**Table 2** shows the demographics of stakeholders involved in water usage and treatment, and/or distribution, and selling water treatment products. We interviewed women (n = 95), water point owners (n = 38), health facility workers (n = 35) and shopkeepers of pharmacy attendants (n = 32). Most of the respondents (n = 131) were female. Over half of the stakeholders (n = 105) were 30–49 years old. The majority had secondary-level education (n = 73), a diploma (n = 56) or a bachelor (n = 35). Their occupations included skilled professions (n = 75) such as traders, hairdressers, tailors, elementary occupations such as selling assorted items (n = 58), and professional occupations such as health workers and pharmacists (n = 53). Regarding water treatment, most respondents (n = 67) reported not treating their water. Close to half of the women and water point owners (n = 60) had heard about chlorine, but very few (n = 13) had experience using it. Despite the scarce knowledge and use of chlorine, most pharmacists (n = 21) reported selling chlorine even when the demand was low.

**Table 2:** Demographics of stakeholders who participated in the in-depth interviews*

Demographics	Total (n = 200), n (%)
Stakeholder type	
*Women*	95 (47.5)
*Health facility workers*	35 (17.5)
*Shopkeepers/pharmacy attendants*	32 (16.0)
*Water point owners/managers*	38 (19.0)
Gender	
*Female*	131 (65.5)
*Male*	69 (34.5)
Age in years	
*<18*	62 (31.0)
*18–29*	105 (52.5)
*30–49*	30 (15.0)
*>50*	3 (1.5)
Level of education	
*No schooling*	1 (0.5)
*Primary*	22 (11.0)
*Secondary*	73 (36.5)
*Bachelors*	35 (17.5)
*Masters*	6 (3.0)
*Diploma*	56 (28.0)
*Missing*	7 (3.5)
Occupation	
*Student*	0 (0.0)
*Unemployed*	8 (4.0)
*Elementary occupations*	58 (29.0)
*Skilled occupations*	75 (37.5)
*Professional occupations*	53 (26.5)
*Missing*	6 (3.0)
Water treatment (women and water point owners)	148 (100)
*Boiling*	16 (10.8)
*Alum*	5 (3.4)
*Letting it settle*	7 (4.7)
*Chlorine*	13 (8.8)
*Filtration*	9 (6.1)
*Reverse osmosis and UV filtration*	2 (1.4)
*Cleaning the tanks*	9 (6.1)
Water treatment (mostly water point owners)	
*Sterilising the cover of container/tank*	2 (1.4)
*Adding unknown chemical*	5 (3.4)
*Water treatment plant/water cooperation*	2 (1.4)
*Treatment is done, but method not mentioned*	2 (1.4)
*None*	67 (45.3)
*Missing*	9 (6.1)
Knowledge about chlorine (women and water point owners)	133 (100)
*Yes*	60 (45.1)
*No*	58 (43.6)
*Missing*	15 (11.3)
Ever sold chlorine (pharmacists)	31 (100)
*Yes*	21 (65.6)
*No*	11 (34.4)
*Missing*	0 (0)

*Number of observations are reported. Proportions are reported in parenthesis. Occupational groups are based on the International Classification of Occupations published by ILO in 2008 [37]. Missing figures are reported for each category when present.

### Main results

We identified the themes around safe water practices and potential interventions – water culture, improving the quality of life, getting support, and advocating for safe water.

### Water culture

The knowledge about the need to treat drinking water was low among women. Most women did nothing to treat their water because they thought it was already safe and could not get re-contaminated when stored in cleaned and previously washed containers. When asked to rank water quality from one to 10, their perceptions were very high across all three states on a scale of eight or more. In particular, women thought water from sachets, community boreholes, or wells was safe because it appeared clean. If they had to treat it when it was dirty, they did that by boiling, using alum or cloth filtering. When asked about chlorine specifically, some women mentioned they knew what chlorine was, mainly in Lagos state, but knowledge and usage were limited. Most women in Kano and Ogun did not know what chlorine was. Women in urban LGAs tended to know more about chlorine than those in rural LGAs. However, the majority did not use chlorine because they considered their water already safe to drink and of high quality. Few women also mentioned disliking the taste or smell of chlorinated water when they tried it, but they would be willing to try and use it again. The knowledge, practices, and beliefs regarding safe drinking water, treatment, and chlorine specifically were similar to those of other stakeholders (**Table 3**).

**Table 3:** Sub-themes that described opinions from participants on water culture

Sub-themes	Quotes
Knowledge	Need for water treatment: ‘People in the community believe they have clean water, so they don’t treat it.’ – male, water point owner, ≥50 y old, Abeokuta South, Ogun.
	Knowledge about chlorine: ‘I don’t treat my water, and no, I don’t [about chlorine].’ – woman with children aged <5 y, 30–49 y old, Tarauni, Kano.
Practices	Use of chlorine: ‘We have been hearing about it, but have never used it.’ – woman, age not reported, Surulere, Lagos.
	[When shown a picture of a bottle of chlorine]: ‘…maybe it is because we don’t really know the value, we might have heard it being mentioned but do not know how it really works. I just think it is just chemical.’ – woman, age not reported, Surulere, Lagos.
	‘I don’t know how it works, that’s why I don’t use it.’ – woman, age not reported, Surulere, Lagos.
Beliefs	Water safety: ‘No we don’t treat the water because the water is transparent, clean and safe for use.’ – male, water point owner, 30–49 y old, Kano municipal, Kano.
	Water quality: ‘I would rate the quality of the water 10, the water is very clean because our supplier uses transparent tanks.’ – woman, 30–49 y old, Ewekoro, Ogun.
	Concerns around chlorine taste and smell: ‘Yes, I used chlorine in the past, didn’t like the taste and smell but will not mind trying it again.’ – woman, 30–49 y old, Yaba, Lagos.
	‘It’s only the smell that changes, but it is ok to me.’ – woman, 30–49 y old, Tarauni, Kano.

### Improving the quality of lives

Only 14% of women in one LGA in Lagos mentioned that their children aged <5 years had diarrhoea in the past two weeks. The proportion of women reporting diarrhoea of their children is higher in Kano and Ogun state, being as low as about 7% in one urban LGA in Ogun to about 71% in an urban LGA in Kano. The most common explanation provided was teething, as children would put unsanitary materials in their mouths, leading to infections and then diarrhoea. A few mentioned unsafe water, and this belief was supported by other stakeholders, such as water point owners. Health workers, instead, especially in urban LGAs, reported diarrhoea to be a rampant problem in their communities, mainly during the rainy season. Many women across states reported incidences of child death, miscarriage, or stillbirth but did not agree that water-related diseases could have contributed to these events. Yet, while most women reported exclusive breastfeeding for the first six months of the child as recommended by the World Health Organization (WHO), some mentioned they would supplement breastmilk with (potentially contaminated) water before that child’s age (**Table 4**).

**Table 4:** Sub-themes that describe opinions from participants on improving the quality of lives

Sub-themes	Quotes
<5 children’s health	Diarrhoea and its reasons: ‘...diarrhoea cases in our facility are seasonal, most especially in January, it then stops in February…’ – female, health facility worker, 30–49 y old, Ado Odo Ota, Ogun.
	‘The cases of diarrhoea I have seen are related to bad environments, no good source of water. We hardly have cases of diarrhoea at the health centre. During the periods we have (cases), we advise that they use Water Guard and we treat them with oral rehydration therapy.’ – female, health facility worker, 30–49 y old, Abeokuta North, Ogun.
	‘Yes, my two-year-old is presently having diarrhoea. My five year old has recurrent diarrhoea. My two-year-old child has had diarrhoea for about two days now, three times in a day…Yes, he has watery diarrhoea and is always very thirsty…Yes, I am concerned.’ – woman, 30–49 y old, Kano municipal, Kano.
	‘...well I don't think the diarrhoea cases are related to unsafe water, rather related to the teething period of the baby.’ – male, water point owner, 30–49 y old, Tarauni, Kano.
	‘...no, they have not experienced diarrhoea in the last two weeks, they did not have any symptoms. I was concerned the last time when the youngest was having diarrhoea. It was not common, only when he was about to produce a tooth. Since he is teething, I only give zinc and oral rehydration therapy in cases of diarrhoea and sometimes traditional medicine. Yes, the reason for diarrhoea was just teething…’ – woman, 18–29 y old, Kura, Kano.
Women’s well-being	Pregnancies and breastfeeding: [woman experienced seven pregnancies] ‘…reason for miscarriage was stooling badly (up to 10 times a day) during first pregnancy and decided to self-medicate with Flagyl medicine. A month after that experience, I woke up to see spotting. When I was taken to the hospital and asked to describe experiences so far, the doctor mentioned self-medication to be the cause of the miscarriage…At the time of the stooling, I was also vomiting…[when asked about whether unsafe water contributed to it]...probably. Now that I think about it but not entirely sure because I drink safe water. Perhaps food prepared from outside was the reason…’ – woman, 30–49 y old, Ijede, Lagos.
	‘Yes, I experienced a miscarriage. Not sure exactly what was the cause of it. I did not have so much knowledge and so I didn’t do things right. Stress could have been a factor as well. I am not sure there is more to tell. No, I do not think miscarriage can be linked to water-borne disease…’ – woman, 30–49 y old, Surulele, Lagos.
	‘...at birth, I gave Zam Zam water (Islamic Holy Water) and then breastmilk. Yes, I breastfed the baby up to 22 mo. I started giving normal borehole water at two months and food at three months. He eats anything…’ – woman, 18–29 y old, Kura, Kano.
	‘...I have two kids and I did exclusive breastfeeding. I was breastfeeding, but my mother insisted that I add water because their throat could be dry, so I started giving her water…’ –woman, years not reported, Surulele, Ijede, Lagos.

### Getting support

Stakeholders reported several issues concerning water availability in their communities, including lack of access to tap water, environmental issues like boreholes or wells drying up, and irregular supply of electricity to pump water from boreholes being the most common problems. They all expressed the need for more support from the government to build a better water infrastructure. However, women, health workers and water point owners agreed that interventions aimed at improving access to safe water treatment would benefit their communities. Health workers supported a programme run through PHCs as pregnant women and children at higher risk would benefit more as already connected. Shopkeepers and pharmacy attendants were willing to support chlorine distribution (**Table 5**).

**Table 5:** Sub-themes that describe opinions from participants on getting support

Sub-themes	Quotes
Government interventions	Water infrastructure: ‘There are not enough boreholes in the community, electricity is bad, plus the price of fuel too. It is always hard to get water.’ – woman, 30–49 y old, Abeokuta North, Ogun.
	‘Government should provide tap water and enough boreholes to the community. Yes, boreholes and wells.’ – female, health worker, 30–49 y old, Ado Odo Ota, Ogun.
	Chlorine distribution: ‘Yes, there was in 2021, the ministry of health came to distribute water guards after a severe diarrhoea outbreak. Aside from the cholera outbreak in 2021, when the government came to our rescue and we were provided WaterGuard to be put in our water, there's been nothing since then.’ – female, health facility worker, female, 30–49 y old, Abeokuta North, Ogun.
	‘...yes, if we can get the products [refer to chlorine], we will be selling it. There was no government project here before.’ – male, pharmacist, 30–49 y old, Ado Odo Ota, Ogun.
Partnerships between private actors, government, and communities	Role of health facilities: ‘If taken outside of the health facilities, the pregnant women and those with children under the age of five may not necessarily get involved in the program, and they need it more than anyone else.’ – female, health worker, ≥50 y old, Kano municipal, Kano.
	‘Primary health centres will be better. There should always be availability of this chlorine for them…[it is important to] provide education on safe water treatments and provide electricity to make water more available to everyone. And chlorine should always be there so they would not get disappointed any time they collect chlorine.’ – female, health worker, 30–49 y old, Ado Odo Ota, Ogun.
	Role of pharmacists: ‘I used to sell it, there is no demand for this product [refer to WaterGuard]. I never heard about a complaint about the product, they used it because it purifies water and makes it safe for drinking. People that have been prescribed in the hospital, sometimes come in a car and ask for it. Yes, I work with health workers like giving out mosquito nets, COVID-19 vaccine, polio vaccine.’ – male, shopkeeper, male, 18–29 y old, Tarauni, Kano.
	Interest of women: ‘All of us here will have no fear or doubt about using chlorine for water treatment because you have just opened our eyes to its usefulness, we will also do the same to other women…Health facilities would be better than any other place’ – woman, years not reported, Kano Municipal, Kano.
	‘...if the Ministry of Health accepts it, then people will undoubtedly accept it too. Yes, just like the polio vaccine, some people will accept and some will dislike it no matter how hard you try to explain it to them.’ – woman, years not reported, Kano Municipal, Kano.
	‘Once you come and educate them and share it with them, most of them will be interested and will go and pick up at the health centre, churches and other places.’ – woman, years not reported, Ijede, Lagos.
	Water point owners: ‘Need for training of the community on the use of the product. If the program (free) cannot continue, let them introduce an affordable payment. If they introduce price/payment in the future, this will ensure the continuity of the program. Some people may not use it and not visit the hospital, so church or mosque could be of use.’ – water point owner, sex and years not reported, Ijiede, Lagos.
	‘Yes, it can be successful. They should make public enlightenment. The distribution should be given only to health centres.’ – male, water point owner, ≥50 y old, Surulele, Ogun.

### Advocating for safe water

All stakeholders agreed that, for a programme to be successful, it would be key to create awareness about safe water treatment, including chlorine, and its benefits. It would be important to create interest not only by women but also by men. The involvement of religious leaders such as ward heads and Imams was also mentioned to ensure that the community could trust the information. There was a general interest in safe water interventions among all stakeholders (**Table 6**).

**Table 6:** Sub-themes that describe opinions from participants on advocating for safe water

Sub-themes	Quotes
Introducing safe water treatment and creating awareness	Education: ‘If people know the value of it, why not, just teach them how to use it, that's all.’ –woman, 18–29 y old, Yaba, Lagos.
	‘Well, you can educate the community on the importance of safe water, also by improving the water source…Yes, I would participate, at least to provide awareness: what is the product, how it is used, how to distribute and the importance…’ – male, health worker, 30–49 y old Tarauni, Kano.
	‘I think [the problem is] lack of education and enlightenment, I think they will use it [refer to chlorine] because not everyone can afford clean water…’ – woman, between 30–49 y old, Surulele, Lagos.
	‘...organising events to educate people on the importance of drinking clean water will benefit people, will be effective, and protect them.’ –male, water point owner, ≥50 y old, Abeokuta South, Ogun.
Integrating services/institution collaboration	Interest of stakeholders: ‘Government should build boreholes for the community. Yes I would fully participate [in the program]. Yes, the program will benefit the people in this community and change their water treatment habits; it will be very effective and will reduce the number of children falling sick due to diarrhoea and coming to the hospital too often.’ – female, health worker, female, 30–49 y old, Ado Odo Ota, Ogun.
	[When asked about distribution of chlorine products] ‘...yes, local pharmacies, shops, mosques, churches, schools, markets.’ – woman, 30–49 y old, Yaba, Lagos.
	‘Of course, I will give out chlorine. Yes, it will, it should! [refer to increasing patronage of his shops if WaterGuard is more available to him without stress].’ – pharmacist, 18–29 y old, Yaba, Lagos.
	‘Yes, [I would give out chlorine], I would even advise people to use things like that. I will give it out for free. If people can get it provided it’s free, it will increase my patronage.’ – shop owner, 18–29 y old, Ewekoro, Ogun.
	‘Health facilities, but also ward-heads and Imams (religious leaders) could be contacted for awareness campaigns. Lack of awareness could hinder proper acceptance of the program.’ – female, health worker, female, ≥50 y old, Kano Municipal, Kano.
	‘As the chairman, there is need for sensitisation...we will inform the CDAs (community development associations), we have over 40 CDAs. It will benefit them, water is the first thing in one’s life. It is about enlightening them.’ – male, water point owner, ≥50 y old, Abeokuta South, Ogun.

## DISCUSSION

### Barriers and facilitators to implementing safe water treatment practices

Following the findings of the four themes and sub-themes, our study identified barriers and facilitators to implementing safe water practices. In terms of barriers, stakeholders first identified a lack of government investment in water infrastructure. Several stakeholders reported the need to improve water access by building more community boreholes. They also mentioned that more government action is needed in normal times instead of primarily responding when water-related disease outbreaks happen.

A second barrier relates to beliefs around water and current existing water practices. Most stakeholders reported that water in their communities was considered safe and high-quality. Stakeholders did not think it was necessary to treat drinking water, especially from boreholes or wells, because it was clean. Despite the positive beliefs held by the community about the cleanliness and safety of their water, the *Escherichia coli* testing we conducted in Ogun state revealed that the water was contaminated. Thirty-four water samples were collected by the research assistants in four LGAs and tested by two senior professors at the Federal University of Agriculture in Ogun. Of the 34 water samples, 12 were samples of stored drinking water, while the remaining 22 samples were obtained from water sources mentioned by women during their IDIs. All the *Escherichia coli* water tests conducted indicated contamination levels surpassing the WHO’s recommended standards, where a zero count of *Escherichia coli* per 100 ml of water is considered safe for drinking. Instead, we noted contamination levels high enough to classify water from all sources (stored or otherwise) as high-risk, with just one sample from among the 34 obtained from water sources being medium-risk [38]. Third, respondents who knew about chlorine or used it before shared concerns about potential taste and smell. Yet, the majority would be willing to try it again once they understood its value and benefits.

A final barrier to the acceptability and feasibility of interventions was access to and affordability of chlorine products. While some respondents knew and used products such as WaterGuard Plus as part of government or non-government organisations’ delivery during emergencies or specific projects, the products are not always available at shops or pharmacies because of low demand. Stakeholders suggested providing the product for free or at an affordable price to ensure enough demand was generated.

In terms of facilitators, first, there was a need for awareness and education to address some of the beliefs mentioned above. The priority is educating and enlightening communities about the importance of safe drinking water, the possibility of re-contamination, and the potential consequences for maternal and child morbidity and mortality. Women who understood the value and potential benefits of point-of-use chlorination during the study reported being willing to try chlorine.

A second facilitator identified from the analysis was the acceptability of stakeholders to the proposed intervention, such as the distribution of point-of-use chlorination products (e.g. WaterGuard Plus). Conditional on making communities aware of the importance, there was an interest across stakeholders about the proposed interventions. Yet, all stakeholders suggested the need for integrating services, especially with the health facilities and with the support of local leaders.

A third facilitator was effective community engagement and close partnerships between the government and the private sector. Specifically, health workers shared that they would be interested in being the platform reaching women and young children and that pharmacists would be willing to sell or distribute the chlorine products for free. Local leaders’ involvement in awareness campaigns and churches, mosques, schools or markets for distribution were some of the proposals given by the stakeholders interviewed. This confirmed the need for a buy-in across all stakeholders participating in this study and beyond.

Altogether, our study contributes to the literature on households’ water treatment practices in African countries [8] by shedding light on the facilitators and barriers of point-of-use chlorination. Building on existing evidence mainly from Uganda [14], we also adopt the socio-ecological model of health to explore the setting of Nigeria.

### Limitations and strengths

In this qualitative study, we utilised FGDs and IDIs to elicit first-hand experiences from different stakeholders who are the end-users of water and water treatment. The study has several limitations. First, the findings were derived from a sample of pregnant women and mothers of children aged <5 years, health facility workers, shopkeepers or pharmacy attendants, and water point owners who are a subset of the potential participants in the country. Second, water practices were identified as practices from communities in Kano, Lagos, and Ogun states, representing only three geopolitical zones and not all six geopolitical zones in Nigeria. Third, we gathered interest in possible interventions providing point-of-use chlorination, and we did not discuss other potential solutions (e.g. investment in water infrastructure) with respondents. Despite these limitations, this study adds important insights regarding safe water treatment practices and the feasibility and acceptability of safe water interventions, i.e. providing point-of-use chlorination, in a country such as Nigeria where more progress needs to be made to make sure everyone has access to safely managed water.

## CONCLUSIONS

In March 2023, the United Nations (UN) convened its first all-UN conference on water in half a century to call all governments to accelerate national efforts towards achieving Sustainable Development Goal – clean water and sanitation for all. Yet, this progress still falls short [39], especially in lower-income countries. The findings from this study described that, in Nigeria, practices around safe water treatment were inconsistent due to barriers such as a lack of knowledge about the need to treat water and its potential benefits for pregnant women and young children, the availability of chlorine products, and possible concerns around taste and smell. However, study participants (pregnant women and mothers of children aged <5 years, health facility workers, shopkeepers or pharmacy attendants, and water point owners) were open to accepting interventions such as point-of-use chlorination as long as they were safe, accessible, and affordable. Suggestions on how such interventions should be grounded in awareness first, integrated with health facilities where women and children already receive services, and supported at the community level by local leaders were provided. This study highlighted the potential to improve access to safe water through point-of-use chlorination, thus ameliorating the quality of lives of Nigerian women and young children.

## Additional material


Online Supplementary Document

## References

[1] World Health Organization. 2023. Drinking-water. Available: https://www.who.int/news-room/fact-sheets/detail/drinking-water. Accessed: 26 June 2024.

[2] Crider YS Tsuchiya M Mukundwa M Ray I Pickering AJ Adoption of Point-of-Use Chlorination for Household Drinking Water Treatment: A Systematic Review. Environ Health Perspect. 2023;131:16001. 10.1289/EHP10839 36715546 PMC9885856

[3] Centers for Disease Control and Prevention (CDC). Chlorination Online. 2014. Available: https://www.cdc.gov/healthywater/drinking/public/water_disinfection.html. Accessed:26 June 2024.

[4] Kremer M, Luby SP, Maertens R, Tan B, Więcek W. Water Treatment And Child Mortality: A Meta-Analysis And Cost-effectiveness Analysis. 2023. National Bureau of Economic Research. Available: https://www.nber.org/papers/w30835. Accessed: 4 September 2024.

[5] Geere JA Cortobius M Who carries the weight of water? Fetching water in rural and urban areas and the implications for water security. Water Altern. 2017;10:513–40.

[6] Graham JP Hirai M Kim SS An Analysis of Water Collection Labor among Women and Children in 24 Sub-Saharan African Countries. PLoS One. 2016;11:e0155981. 10.1371/journal.pone.0155981 27248494 PMC4889070

[7] Geremew A Mengistie B Mellor J Lantagne DS Alemayehu E Sahilu G Appropriate household water treatment methods in Ethiopia: household use and associated factors based on 2005, 2011, and 2016 EDHS data. Environ Health Prev Med. 2018;23:46. 10.1186/s12199-018-0737-9 30261840 PMC6161466

[8] Geremew A Damtew YT Household water treatment using adequate methods in sub-Saharan countries: evidence from 2013–2016 demographic and health surveys. J Water Sanit Hyg Dev. 2020;10:66–75. 10.2166/washdev.2019.107

[9] Eticha M Geremew A Dirirsa G Bayu K Girma H Mengistu DA Household water treatment practice and associated factors among households dependent on unimproved water sources in Ameya district, Oromia, Ethiopia. J Water Sanit Hyg Dev. 2022;12:432–42. 10.2166/washdev.2022.034

[10] Asefa L Ashenafi A Dhengesu D Roba H Lemma H Household water treatment practice and associated factors among rural Kebeles (villages) in west Guji zone, southern Ethiopia: Community based cross-sectional study. Clin Epidemiol Glob Health. 2023;22:101311. 10.1016/j.cegh.2023.101311

[11] Desye B Tesfaye AH Berihun G Sisay T Daba C Berhanu L Household water treatment practice and associated factors in Ethiopia: A systematic review and meta-analysis. PLoS One. 2023;18:e0285794. 10.1371/journal.pone.0285794 37289814 PMC10249828

[12] Tamene A A Qualitative Analysis of Factors Influencing Household Water Treatment Practices Among Consumers of Self-Supplied Water in Rural Ethiopia. Risk Manag Healthc Policy. 2021;14:1129–39. 10.2147/RMHP.S299671 33758565 PMC7981144

[13] Mitro B Wolfe MK Galeano M Sikder M Gallandat K Lantagne D Barriers and facilitators to chlorine tablet distribution and use in emergencies: a qualitative assessment. Water. 2019;11:1121. 10.3390/w11061121

[14] Ssemugabo C Halage AA Namata C Musoke D Ssempebwa JC A socio-ecological perspective of the facilitators and barriers to uptake of water, sanitation and hygiene interventions in a slum setting in Kampala, Uganda: A qualitative study. J Water Sanit Hyg Dev. 2020;10:227–37. 10.2166/washdev.2020.124

[15] Alam MU Unicomb L Ahasan SM Amin N Biswas D Ferdous S Barriers and enabling factors for central and household level water treatment in a refugee setting: a mixed-method study among Rohingyas in Cox’s Bazar, Bangladesh. Water. 2020;12:3149. 10.3390/w12113149

[16] Tseole NP Mindu T Kalinda C Chimbari MJ Barriers and facilitators to Water, Sanitation and Hygiene (WaSH) practices in Southern Africa: A scoping review. PLoS One. 2022;17:e0271726. 10.1371/journal.pone.0271726 35917339 PMC9345477

[17] Clasen T Roberts I Rabie T Schmidt W Cairncross S Interventions to improve water quality for preventing diarrhoea. Cochrane Database Syst Rev. 2006;CD004794. 10.1002/14651858.CD004794.pub2 16856059

[18] World Health Organization. Results of round II of the WHO international scheme to evaluate household water treatment technologies. 2019. Available: https://www.who.int/publications/i/item/9789241516037. Accessed: 26 June 2024.

[19] Cooper J Examining factors that influence a woman’s search for information about menopause using the socio-ecological model of health promotion. Maturitas. 2018;116:73–8. 10.1016/j.maturitas.2018.07.013 30244782

[20] Ma PHX Chan ZCY Loke AY The Socio-Ecological Model Approach to Understanding Barriers and Facilitators to the Accessing of Health Services by Sex Workers: A Systematic Review. AIDS Behav. 2017;21:2412–38. 10.1007/s10461-017-1818-2 28631228

[21] Ezenwaka U Mbachu C Ezumah N Eze I Agu C Agu I Exploring factors constraining utilization of contraceptive services among adolescents in Southeast Nigeria: an application of the socio-ecological model. BMC Public Health. 2020;20:1162. 10.1186/s12889-020-09276-2 32711497 PMC7382857

[22] Uchendu C Windle R Blake H Perceived Facilitators and Barriers to Nigerian Nurses’ Engagement in Health Promoting Behaviors: A Socio-Ecological Model Approach. Int J Environ Res Public Health. 2020;17:1314. 10.3390/ijerph17041314 32085607 PMC7068510

[23] Salihu HM Wilson RE King LM Marty PJ Whiteman VE Socio-ecological Model as a Framework for Overcoming Barriers and Challenges in Randomized Control Trials in Minority and Underserved Communities. Int J MCH AIDS. 2015;3:85–95. 27621990 PMC4948176

[24] Townsend N Foster C Developing and applying a socio-ecological model to the promotion of healthy eating in the school. Public Health Nutr. 2013;16:1101–8. 10.1017/S1368980011002655 22014870 PMC10271294

[25] Cornelius LJ Erekaha SC Okundaye JN Sam-Agudu NA A Socio-Ecological Examination of Treatment Access, Uptake and Adherence Issues Encountered By HIV-Positive Women in Rural North-Central Nigeria. J Evid Inf Soc Work. 2018;15:38–51. 10.1080/23761407.2017.1397580 29236624

[26] Nyaaba GN Olaleye AO Obiyan MO Walker O Anumba DOC A socio-ecological approach to understanding the factors influencing the uptake of intermittent preventive treatment of malaria in pregnancy (IPTp) in South-Western Nigeria. PLoS One. 2021;16:e0248412. 10.1371/journal.pone.0248412 33720947 PMC7959387

[27] National Bureau of Statistics, United Nations Children’s Fund (UNICEF). Multiple Indicator Cluster Survey 2021, Statistical Snapshot Report. 2022. Available: https://www.unicef.org/nigeria/reports/2021-multiple-indicator-cluster-survey-national-immunization-coverage-survey-report. Accessed: 26 June 2024.

[28] Braun V Clarke V Using thematic analysis in psychology. Qual Res Psychol. 2006;3:77–101. 10.1191/1478088706qp063oa

[29] Clarke V Braun V Thematic analysis. J Posit Psychol. 2017;12:297–8. 10.1080/17439760.2016.1262613

[30] Castleberry A Nolen A Thematic analysis of qualitative research data: Is it as easy as it sounds? Curr Pharm Teach Learn. 2018;10:807–15. 10.1016/j.cptl.2018.03.019 30025784

[31] Kiger ME Varpio L Thematic analysis of qualitative data: AMEE Guide No. 131. Med Teach. 2020;42:846–54. 10.1080/0142159X.2020.1755030 32356468

[32] Bihu R. Qualitative Data Analysis: Novelty in Deductive and Inductive Coding. Advance. 2024. Available: https://advance.sagepub.com/users/717923/articles/705285-qualitative-data-analysis-novelty-in-deductive-and-inductive-coding. Accessed: 4 September 2024.

[33] Wong L Data analysis in qualitative research: a brief guide to using nvivo. Malays Fam Physician. 2008;3:14–20. 25606106 PMC4267019

[34] Vaismoradi M Snelgrove S Theme in qualitative content analysis and thematic analysis. Forum Qual Soc Res. 2019;20:23.

[35] Rivas C. Finding themes in qualitative data. Researching society and culture. 2018. Available: https://us.sagepub.com/en-us/nam/researching-society-and-culture/book248100. Accessed: 4 September 2024.

[36] Cassell C Bishop V Qualitative data analysis: Exploring themes, metaphors and stories. Eur Manag Rev. 2019;16:195–207. 10.1111/emre.12176

[37] International Labour Office. International Standard Classification of Occupations 2008 (ISCO-08): Structure, group definitions and correspondence tables. 2012. Available: https://www.ilo.org/sites/default/files/wcmsp5/groups/public/@dgreports/@dcomm/@publ/documents/publication/wcms_172572.pdf Accessed: 26 June 2024.

[38] Odonkor ST Mahami T Escherichia coli as a Tool for Disease Risk Assessment of Drinking Water Sources. Int J Microbiol. 2020;2020:2534130. 10.1155/2020/2534130 32612658 PMC7313150

[39] Gordon B Boisson S Johnston R Trouba DJ Cumming O Unsafe water, sanitation and hygiene: a persistent health burden. Bull World Health Organ. 2023;101:551–551A. 10.2471/BLT.23.290668 37663869 PMC10452937

