# Supplementary material for: Safe water treatment practices: A qualitative study on point-of-use chlorination in Nigeria
Source: J Glob Health. 2024 Sep 13;14:04178. doi: 10.7189/jogh.14.04178 (PMC11395956; doi:10.7189/jogh.14.04178)
Supplement: Online Supplementary Document [file jogh-14-04178-s001.pdf]

Online Supplementary Document

Table S1. Number of participants, by type and by Local Government Areas (LGA) in Kano, Lagos, Ogun states.

	In Depth Interviews (IDIs)				Focus Group Discussions (FGDs)
LGA	Women	Health workers	Shopkeepers or Pharmacy attendants	Water point owners	Women
**Kano**					
Kura (rural)	9	3	2	4	8
Tarauni (urban)	7	5	3	3	8
Kano Municipal (urban)	10	6	4	2	8
**Lagos**					
Ijede (rural)	8	4	3	3	8
Surulere (urban)	8	3	5	3	8
Yaba (urban)	7	5	2	2	7
**Ogun**					
Abeokuta North (rural)	19	2	3	6	0
Ado Odo Ota (peri-urban)	10	3	4	9	0
Abeokuta South (urban)	13	4	5	6	0
Ewekoro (urban)	4	0	1	0	0
**Total**	**95**	**35**	**32**	**38**	**47**
